# Translation, cross-cultural adaptation, and validation of the Athlete Fear Avoidance Questionnaire (AFAQ) into Brazilian Portuguese

**DOI:** 10.1186/s12891-022-05951-0

**Published:** 2022-11-10

**Authors:** Jodimar Ribeiro dos Reis-Junior, Daniela Bassi-Dibai, Daniel Nunes Morais, André Pontes-Silva, Letícia Padilha Mendes, Jocassia Silva Pinheiro, Cid André Fidelis-de-Paula-Gomes, Almir Vieira Dibai-Filho

**Affiliations:** 1 Department of Physical Education, Universidade Federal do Maranhão, São Luís, MA Brazil; 2 Postgraduate Program in Programs Management and Health Services, Universidade Ceuma, São Luís, MA Brazil; 3 Department of Physical Therapy, Universidade Federal de São Carlos, São Carlos, SP Brazil; 4 Departamento de Fisioterapia, Programa de Pós-Graduação em Fisioterapia, Universidade Federal de São Carlos, Rod. Washington Luís, Km 235, São Carlos, SP CEP 13565-905 Brazil; 5 Postgraduate Program in Rehabilitation Science, Universidade Nove de Julho, São Paulo, SP Brasil; 6 Postgraduate Program in Physical Education, Universidade Federal do Maranhão, São Luís, MA Brazil

**Keywords:** Reproducibility of results, Sports, Surveys and questionnaires

## Abstract

**Background:**

Psychological factors play an important role in the adequate return of an athlete to sport. Our aim was to perform the translation, cross-cultural adaptation, and validation of the Athlete Fear Avoidance Questionnaire (AFAQ) into Brazilian Portuguese.

**Methods:**

We performed the translation and cross-cultural adaptation and evaluated the structural validity, construct validity, and test–retest reliability. In addition to the AFAQ, we used the Numerical Pain Scale (NPS), Pain-Related Catastrophizing Thoughts Scale (PCTS), Self-Estimated Functional Inability because of Pain Questionnaire for athletes (SEFIP-sport), and Hospital Anxiety and Depression Scale (HADS). We used the exploratory factor analysis (EFA) to analyze the internal structure of the AFAQ. We used the Spearman’s correlation coefficient (rho) to determine the magnitude of correlation between the AFAQ and the other instruments. We evaluated the test–retest reliability and internal consistency by means of intraclass correlation coefficient (ICC) and Cronbach’s alpha, respectively.

**Results:**

No adaptation was necessary to produce the AFAQ version in Brazilian Portuguese. We included 160 participants in the study. We identified the one-dimensionality of the AFAQ through the EFA with the implementation of parallel analysis (KMO = 0.83, *p* < 0.001 in Bartlett's Sphericity test). In construct validity, the magnitudes of correlation between the AFAQ and the other instruments ranged from 0.257 to 0.548. We identified adequate reliability (ICC = 0.85) and internal consistency (Cronbach’s alpha = 0.90).

**Conclusion:**

The Brazilian version of the AFAQ with one domain and 10 items has adequate measurement properties in injured professional and recreational athletes.

**Supplementary Information:**

The online version contains supplementary material available at 10.1186/s12891-022-05951-0.

## Introduction

In the rehabilitation process of an injured athlete, several aspects are considered for the return to sport. Among the physical aspects, muscle strength (quadriceps, hamstring, and hip muscles), hop test, and the knee range of motion are commonly considered by physical therapists [1, 2]. In addition, psychological factors (e.g., fear) play an important role in the adequate return of an athlete to sport [3]. Studies support that fear is a prominent emotional response at the time of recovery from injury [4]. However, in this scenario, few questionnaires or scales were created and validated to assess the fear of returning to sport [5, 6].

The specialized scientific literature presents two instruments to measure the fear of returning to sport: Anterior Cruciate Ligament – Return to Sport after Injury (ACL-RSI), created in the English language [7] and validated for Brazilians by Silva et al. [5]; and Athlete Fear Avoidance Questionnaire (AFAQ), developed by Dover and Amar [6] in the English language for Canadians, composed of ten items investigating the fear of returning to sport (in team sports athletes’ injured).

Furthermore, the AFAQ also features a wide scope (due to its possibility of use in different age groups) and practicality (reduced number of items, small possibility of redundant items, small possibility of errors, and shorter filling time). The original version of the AFAQ found measurement properties suitable for the instrument [6]. To date, only one cross-cultural adaptation of the AFAQ is described in the literature, a study conducted by Monticone et al. [8] showing adequate measurement properties for Italians. Therefore, we aimed to perform the translation, cross-cultural adaptation, and validation of the AFAQ into Brazilian Portuguese.

## Methods

### Study design

This is a questionnaire validation study carried out according to the Guidelines for the Process of Cross-cultural Adaptation of Self-Report Measures [9] and Consensus-based Standards for the Selection of Health Measurement Instruments (COSMIN) [10]. The authorization to carry out the adaptation of the AFAQ to Brazilian Portuguese was granted via e-mail by one of the authors of the questionnaire (Dr. Geoffrey Dover). We carried the study out in three phases: 1) translation and cross-cultural adaptation, 2) test of the pre-final version of the AFAQ into Brazilian Portuguese, and 3) validation of the final version of the AFAQ cross-culturally adapted to Brazilian Portuguese.

We have performed data collection through the free Google Forms platform (Mountain View, CA, USA). Study procedures were approved by the institution’s Research Ethics Committee (report number 4,256,651). We disseminated the research on social media and physical therapy clinics.

### Participants

We calculated the sample size according to the COSMIN recommendation of 7 times the number of items in the questionnaire provided that this value is not lower than 100 participants [10]. We considered the following inclusion criteria: recreational (individual who is physically active but who does not train for competition [11]) or professional athletes born in Brazil, away from any type of team sport for at least 7 days, aged 18 years or older, and literate. Exclusion criteria: participants who do not wish to return to sport, as well as those presenting any cognitive impairment or severe anxiety or depression.

### Translation and cross-cultural adaptation

The process of translation and cross-cultural adaptation of the AFAQ into Brazilian Portuguese was in accordance with study conducted by Beaton et al. [9].

- 1) Translation: two independent translators (a physical therapist with 10 years of experience in the field and an English teacher with 20 years of experience in translation without technical knowledge in the health area), performed the translation of the original version of the AFAQ into Brazilian Portuguese. Both have Brazilian Portuguese as their mother tongue and are fluent in English.
- 2) Synthesis of the translations: after discussions and revisions, both translators, under observation of one of the researchers, synthesized the two versions of the translated questionnaire and produced a single version of the AFAQ in a consensual way.
- 3) Back-translation: two independent translators (without technical knowledge in the health area), both having English as their mother tongue and fluent in Portuguese, carried out the translation of the Portuguese version of the AFAQ back into English, without no prior knowledge of the original version of the questionnaire.
- 4) Analysis by a committee of experts: 4 experts from the rehabilitation area, together with the 4 translators involved in the project, reviewed all translated and back-translated versions to correct possible discrepancies, thus achieving the pre-final version of the AFAQ in a way agreed among all the members of the committee.
- 5) Test of the pre-final version: we applied the pre-final version of the AFAQ to 30 injured athletes who have Brazilian Portuguese as their mother tongue. Participants read and filled out the questionnaire and, at the end of completion, demonstrated their understanding of the pre-final version of the AFAQ by ticking a checkbox containing the “yes” and “no” answers for each item of the questionnaire. If there were items that could not be understood by more than 20% of the participants, they should be reformulated and retested on a new sample of 30 participants [12], until the desired level of understanding was reached.

### Assessment of measurement properties

After defining the final version of the AFAQ, we evaluated the following measurement properties: structural validity, construct validity, and test–retest reliability. For this, we performed two applications of the AFAQ with an interval of 1 week between them. We defined this time interval based on COSMIN [10] and previous reliability studies [11, 13], since the time interval should be long enough to prevent recall bias, and short enough to ensure that patients have not been changed on the construct to be measured [10]. At first, we applied the AFAQ and the following instruments to validate the construct: the Numerical Pain Scale (NPS), Pain-Related Catastrophizing Thoughts Scale (PCTS), Self-Estimated Functional Inability because of Pain Questionnaire for athletes (SEFIP-sport), and Hospital Anxiety and Depression Scale (HADS). We performed the second application of the AFAQ to be able to measure the test–retest reliability.

Regarding the instruments used here, the AFAQ is a questionnaire containing 10 items and 5 options of answers: 1 – Not at all; 2 – To a slight degree; 3 – To a moderate degree; 4 – To a great degree; 5 – Completely agree. To reach the final score, the sum of all the marked answers must be performed, generating a score that varies from 10 to 50. Higher values indicate greater fear of returning to sport [6].

The NPS measures pain intensity through a sequence of 11 numbers (from 0 to 10), so that 0 represents “no pain” and 10 indicates “maximum imaginable pain”. A study conducted by Ferreira-Valente et al. [14] validated this scale for Portuguese.

The PCTS was validated for Brazil by Sardá-Junior et al. [15] and it measures catastrophic thoughts through 9 items. To calculate the total score, all items must be added and divided by the answered items, generating a value that varies from 0 to 5. Higher scores indicate greater catastrophizing.

The SEFIP-sport was validated for Brazil by Reis-Junior et al. [16] and Reis-Junior et al. [17] and it measures disability related to sports practice through 14 items. To calculate the total score, all the values related to the answers must be added, generating a value that varies from 0 to 56 points. Higher values indicate greater disability.

The HADS was validated for Brazil by Castro et al. [18] and it measures symptoms of anxiety and depression through 14 items (7 items for anxiety and 7 items for depression). To calculate the score by domain, the answered items must be added, generating a value that varies from 0 to 21. Higher values indicate greater symptoms.

### Statistical analysis

We performed descriptive statistics and the variables were presented as mean and standard deviation (SD) or absolute and relative frequency. We used the SPSS software (version 17.0, Chicago, IL, USA) for the analyses of descriptive statistics, reliability, internal consistency, and construct validity.

We used the exploratory factor analysis (EFA) to analyze the internal structure of the AFAQ. We used the implementation of a polychoric matrix and a robust diagonally weighted least squares (RDWLS) extraction method, since the response possibilities for each AFAQ item are ordinal values [19, 20]. We defined the identification of the number of factors to be retained by means of parallel analysis with random permutation of the observed data via robust promin [21, 22]. We performed the data processing using the FACTOR software (Universitat Rovira i Virgili, Tarragona, Spain). We assessed the model adequacy using the Kaiser–Meyer–Olkin (KMO) criterion and Bartlett's Sphericity test. A KMO value above 0.70 and a significant *p* value (< 0.05) in the Bartlett test are considered adequate indices [23, 24].

In addition, we calculated internal consistency using Cronbach’s alpha to identify whether there are redundant or heterogeneous items in the questionnaire. We considered adequate value on Cronbach’s alpha > 0.70 [10]. We evaluated the reliability based on a test–retest model, using the intraclass correlation coefficient (ICC). We considered adequate value on ICC > 0.75 [25]. In addition, we calculated the standard error of measurement (SEM), minimum detectable change (MDC), and coefficient of variation (CV) [26].

On the construct validity, we used Spearman's correlation coefficient (rho) to determine the magnitude of correlation between the AFAQ and the other instruments. As there is no instrument with a similar construct used in Brazil, our hypothesis is that correlations with instruments that measure related but different constructs should range from 0.30 to 0.50 [10].

Ceiling and floor effects have been evaluated in this study. By definition, these effects occur when more than 15% of the study participants reach the minimum or maximum values ​​of the questionnaire as a total score.

## Results

### Translation and cross-cultural adaptation

Regarding translation and cross-cultural adaptation, there was consensus among translators and experts and no adaptation or significant change was necessary to produce the AFAQ version in Brazilian Portuguese, keeping the sense of the items of the original version, including items related to team sport (e.g., item 2 – “I am worried about my role with the team changing”).

In the pre-test phase, we included 32 participants. Of these individuals, 24 (75%) is male and had a mean age of 24.91 years (SD = 4.82), and 8 (25%) is female and had a mean age of 27.38 years (SD = 8.38). The most practiced sports were: soccer (*n* = 13, 40.62%), basketball (*n* = 8, 25%), futsal (*n* = 5, 15.62%), volleyball (*n* = 4, 12.5%) and handball (*n* = 2, 6.25%). All items of the AFAQ were 100% understood by the 32 athletes. Thus, we have established the final version of the Brazilian version of the AFAQ (Additional file 1).

### Sample characteristics

We included 160 participants in the study. Most of the sample is composed of men, young adults, single, and with incomplete higher education, as shown in Table 1. Regarding the characteristics related to sport and injury, as shown in Table 2, most of the sample played football, with an average weekly frequency of more than 3 times and with a total time of practice in the sport of more than 112 months. Regarding the injury, ACL rupture and sprain were the most common injuries, affecting mainly the knee and ankle.

**Table 1:** Personal characteristics of the study sample (*n* = 160)

**Characteristics**	**Mean (standard deviation) or n (%)**
Age (years)	31.3 (11)
Sex	
Male	105 (65.6%)
Female	55 (34.4%)
Weight (kg)	75.3 (14.4)
Height (m)	1.7 (0.1)
Body mass index (kg/m^2^)	25.1 (4.1)
Marital status	
Single	117 (73.1%)
Married	40 (25%)
Divorced	1 (0.6%)
Widower	2 (1.3%)
Level of education	
Complete primary education	1 (0.6%)
Incomplete secondary education	3 (1.9%)
Complete secondary education	21 (13.1%)
Incomplete higher education	61 (38.1%)
Complete higher education	25 (15.6%)
Incomplete postgraduate	17 (10.6%)
Complete postgraduate	32 (20%)
NPS (score, 0–10)	3.9 (2.8)
PCTS (score, 0–5)	1.1 (1.1)
SEFIP-sport (score, 0–56)	5.8 (4.6)
HADS	
Anxiety (score, 0–21)	7.1 (4.2)
Depression (score,0–21)	4.7 (3.5)
AFAQ (score, 10–50)	27.6 (9.1)

*NPS* Numerical Pain Scale, *PCTS* Pain-Related Catastrophizing Thoughts Scale, *SEFIP-sport* Self-Estimated Functional Inability because of Pain Questionnaire for athletes, *HADS* Hospital Anxiety and Depression Scale, *AFAQ* Athlete Fear Avoidance Questionnaire

**Table 2:** Clinical and sports characterization of the study sample (*n* = 160)

**Characteristics**	**Mean (standard deviation) or n (%)**
Sport modality	
Football	77 (48.1%)
Volleyball	27 (16.9%)
Futsal	17 (10.6%)
Basketball	17 (10.6%)
Handball	17 (10.6%)
Rugby	3 (1.9%)
American football	2 (1.3%)
Weekly frequency (times)	3.9 (1.4)
Weekly practice time (minutes)	333.3 (247.2)
Total practice time (months)	112.9 (220.5)
Injury time (months)	6.8 (8.6)
Injury	
ACL rupture	30 (18.8%)
Sprain	29 (18.1%)
Fracture	13 (8.1%)
Torn meniscus	13 (8.1%)
Tendinitis	13 (8.1%)
Muscle tear	11 (6.9%)
Non-specific pain	10 (6.3%)
Patellofemoral pain	7 (4.4%)
Herniated disc	6 (3.8%)
Dislocation	6 (3.8%)
Rupture of other ligaments	6 (3.8%)
Plantar fasciitis	3 (1.9%)
Others	10 (6.3%)
Injury site	
Knee	68 (42.5%)
Ankle	31 (19.4%)
Shoulder	13 (8.1%)
Lower back	8 (5%)
Foot	7 (4.4%)
Thigh	6 (3.8%)
Leg	5 (3.1%)
Elbow	4 (2.5%)
Arm	3 (1.9%)
Wrist	3 (1.9%)
Others	12 (7.5%)

*ACL* Anterior cruciate ligament

### Structural validity

**Fig. 1:**
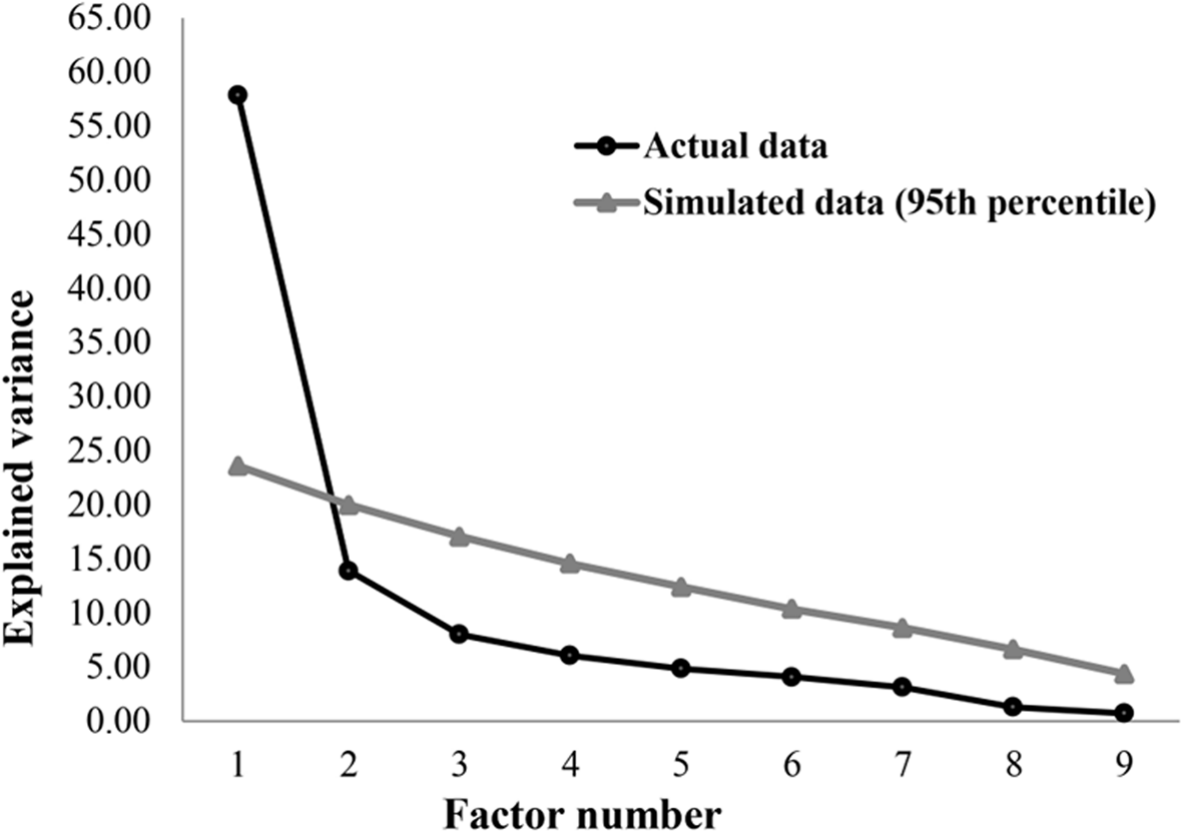
Scree plot of the parallel analysis demonstrating the one-dimensionality of the Athlete Fear Avoidance Questionnaire (AFAQ)

We identified the one-dimensionality of the AFAQ through the EFA with the implementation of parallel analysis (KMO = 0.83, *p* < 0.001 in Bartlett's Sphericity test), as shown in Fig. 1. The appropriate factor loadings (> 0.30) of each AFAQ item are described in Table 3.

**Table 3:** Factor loadings of the Athlete Fear Avoidance Questionnaire (AFAQ)

**Item**	**Factor loading (90% confidence interval)**
1	0.708 (0.607, 0.780)
2	0.607 (0.448, 0.691)
3	0.626 (0.505, 0.709)
4	0.352 (0.200, 0.490)
5	0.782 (0.707, 0.836)
6	0.751 (0.652, 0.824)
7	0.728 (0.624, 0.797)
8	0.799 (0.704, 0.855)
9	0.781 (0.695, 0.844)
10	0.693 (0.589, 0.772)

### Construct validity

As previously defined in the hypothesis and described in Table 4, the magnitudes of correlation between the AFAQ and the other instruments ranged from 0.257 to 0.548, given that they are related instruments, but with different constructs.

**Table 4:** Correlation between the Athlete Fear Avoidance Questionnaire (AFAQ) and the other study instruments

**Instruments**	**AFAQ**
NPS	rho = 0.403, *p* < 0.001 *
PCTS	rho = 0.548, *p* < 0.001 *
SEFIP-sport	rho = 0.228, *p* = 0.004 *
HADS	
Anxiety	rho = 0.399, *p* < 0.001 *
Depression	rho = 0.257, *p* = 0.001 *

*NPS* Numerical Pain Scale, *PCTS* Pain-Related Catastrophizing Thoughts Scale, *SEFIP-sport* Self-Estimated Functional Inability because of Pain Questionnaire for athletes, *HADS* Hospital Anxiety and Depression Scale

^*^ Significant correlation (*p* < 0.05)

### Ceiling and floor effects

We did not observe ceiling and floor effects, as 5 (3.1%) participants reached the minimum score of 10 points and no participant reached the maximum score of 50 points on the AFAQ.

### Reliability and internal consistency

We used a sub-sample (*n* = 37) for reliability and internal consistency analyses. As shown in Table 5, we identified adequate reliability, with ICC = 0.85, SEM = 13.33% and CV = 7.53%. In addition, the AFAQ has adequate internal consistency (Cronbach’s alpha = 0.90).

**Table 5:** Reliability and internal consistency of the Athlete Fear Avoidance Questionnaire (AFAQ)

**Reliability and internal consistency**	**Values**
Test	
Mean	27.32
Standard deviation	8.47
Retest	
Mean	25.83
Standard deviation	8.23
Intraclass correlation coefficient	0.85
95% confidence interval	0.71, 0.92
Standard error of measurement	
Score	3.54
%	13.33
Minimum detectable change	
Score	9.82
%	36.95
Coefficient of variation (%)	7.53
Cronbach’s alpha	0.90

## Discussion

Our study identified that the Brazilian version of the AFAQ has a one-dimensional structure, reliable and with a valid construct, without ceiling and floor effects. To date, only two studies have been published measuring the measurement properties of the AFAQ [6, 8]. Regarding the internal structure, the original [6] and Italian versions [8] identified a one-dimensional structure of the instrument, as in the present study. However, the Italian version highlights that items 6 and 9 presented low factor loadings, suggesting the presence of a small secondary dimension [8].

We identified correlations ranging from 0.257 to 0.548 with the other instruments used in this study. The highest correlations we found were between the AFAQ and pain intensity (rho = 0.403) and catastrophizing (rho = 0.548). The original version of the instrument identified a similar magnitude of correlation with catastrophizing (*r* = 0.587) [6]. Moreover, similarly to the present study, the Italian version of the AFAQ adequately correlated with pain intensity (rho = 0.42) and catastrophizing (rho = 0.59) [8].

Regarding reliability, we identified ICC values of 0.85 and Cronbach’s alpha values of 0.90. The original version of the AFAQ did not calculate test–retest reliability using the ICC, presenting only Cronbach’s alpha value of 0.80 [6]. In the Italian version of the instrument, the authors identified a higher ICC value than the one in the present study (0.95) and a lower Cronbach’s alpha value than the one in the present study (0.78) [8]. However, all values are within acceptability cut-off points [25, 27].

Regarding cross-cultural adaptation, this aspect is an important process to allow questionnaires and scales to be adapted to different cultures and languages without losing evaluative capacity [9]. During the translation of the AFAQ into Brazilian Portuguese, we did not need to adapt any terms to the local culture, respecting the construction of the questionnaire in its original language [6], including the maintenance of items related to team sport (e.g., item 2 – “I am worried about my role with the team changing”).

The Brazilian Portuguese version of the AFAQ provides Brazilian physicians with an essential instrument for use in the clinical setting given its adequate measurement properties in the evaluation of the fear of returning to sport, a clinical variable recognized as important in the rehabilitation of athletes according to a previous systematic review [4]. However, due to the recent creation of the AFAQ, we recommend that future studies define the instrument cut-off point that indicates the high probability of re-injury.

This study has limitations that must be considered. Our collection took place online, thus, the type of injury and the injury region of the body were self-reported (without the clinical diagnosis of a specialized health professional). The collection took place during the COVID-19 pandemic period; therefore, the participants’ psychological condition may have been influenced by this global emergency situation, in addition to the social restriction imposed by this period. We did not assess the phase of the injury or rehabilitation of each participant and this should be considered in the analysis of our results. The AFAQ was translated and adapted cross-culturally in only one different country and this fact minimized the comparison and discussion of the results.

## Conclusion

The Brazilian version of the AFAQ with one domain and 10 items has adequate measurement properties in injured professional and recreational athletes.

## Supplementary Information

**Additional file 1. **Brazilian Version of the Athlete Fear Avoidance Questionnaire (AFAQ).

**Additional file 2. **English Version of the Athlete Fear Avoidance Questionnaire (AFAQ).

## Data Availability

The data and materials in this paper are available from the corresponding author on request.
