# Supplementary material for: Translation, cross-cultural adaptation, and validation of the Athlete Fear Avoidance Questionnaire (AFAQ) into Brazilian Portuguese
Source: BMC Musculoskelet Disord. 2022 Nov 10;23:974. doi: 10.1186/s12891-022-05951-0 (PMC9647760; doi:10.1186/s12891-022-05951-0)
Supplement: Supplementary file 1 — Additional file 1. Brazilian Version of the Athlete Fear Avoidance Questionnaire (AFAQ). [file 12891_2022_5951_MOESM1_ESM.pdf]

Versão Brasileira do Athlete Fear Avoidance Questionnaire (AFAQ)

Nome:
Modalidade esportiva:
Data:

Instruções: Nós estamos interessados nos pensamentos ou sentimentos que você tem quando sente dores relacionadas a lesões esportivas. Usando a escala abaixo, indique, por favor, o grau que representa a presença desses pensamentos e sentimentos quando você sente tais dores.

**Grau**	1	2	3	4	5
**Significado**	Absolutamente nada	Grau leve	Grau moderado	Grau alto	Grau máximo

**Afirmação**	**Grau**
1. Eu nunca mais serei capaz de jogar como fazia antes da minha lesão.	
2. Eu estou preocupado(a) com a minha participação na equipe diante das mudanças que estão ocorrendo nela.	
3. Eu estou preocupado(a) com o que as outras pessoas vão pensar de mim se eu não voltar a atuar no mesmo nível.	
4. Eu não tenho certeza sobre o que é minha lesão.	
5. Eu acredito que a minha lesão atual tenha comprometido minhas futuras habilidades atléticas.	
6. Eu não me sinto confortável em voltar a jogar até estar 100%.	
7. As pessoas não entendem a gravidade da minha lesão.	
8. Eu não sei se estou pronto(a) para jogar.	
9. Eu receio que minha lesão possa se agravar se eu voltar a jogar antes do tempo.	
10. Eu receio que a minha lesão é muito grave quando minha dor é intensa.	
