# Supplementary material for: Translation, cross-cultural adaptation, and validation of the Athlete Fear Avoidance Questionnaire (AFAQ) into Brazilian Portuguese
Source: BMC Musculoskelet Disord. 2022 Nov 10;23:974. doi: 10.1186/s12891-022-05951-0 (PMC9647760; doi:10.1186/s12891-022-05951-0)
Supplement: Supplementary file 2 — Additional file 2. English Version of the Athlete Fear Avoidance Questionnaire (AFAQ). [file 12891_2022_5951_MOESM2_ESM.pdf]

Name: **Sport:** **Date:**

Athletic Fear Avoidance Questionnaire (AFAQ)

Instructions: We are interested in your feelings or thoughts when in pain as a result of a sport injury. Using the following scale, please indicate the degree to which you have these thoughts and feelings when you are in pain due to a sports injury.

**Rating**	1	2	3	4	5
**Meaning**	Not at all	To a slight degree	To a moderate degree	To a great degree	Completely agree

**Statement**	**Rating**
1. I will never be able to play as I did before my injury.	
2. I am worried about my role with the team changing.	
3. I am worried about what other people will think of me if I don't perform at the same level.	
4. I am not sure what my injury is.	
5. I believe that my current injury has jeopardized my future athletic abilities.	
6. I am not comfortable going back to play until I am 100%.	
7. People don't understand how serious my injury is.	
8. I don't know if I am ready to play.	
9. I worry if I go back to play too soon I will make my injury worse.	
10. When my pain is intense, I worry that my injury is a very serious one.	
